# Progressive Familial Intrahepatic Cholestasis Type 1 Associated with Cherry-Red Spots in an Infant: A First Case Report

**DOI:** 10.7759/cureus.12226

**Published:** 2020-12-22

**Authors:** Sarah Alzebaidi, Yara Alghamdi, Amal Alghamdi, Mohammed Hasosah, Emad Alsharef

**Affiliations:** 1 Pediatrics, King Saud bin Abdulaziz University for Health Sciences College of Medicine, Jeddah, SAU; 2 Pediatric Gastroenterology, King Abdullah International Medical Research Center, King Saud bin Abdulaziz University for Health Sciences, Jeddah, SAU; 3 Family Medicine, King Abdullah International Medical Research Center, King Saud bin Abdulaziz University for Health Sciences, Jeddah, SAU

**Keywords:** cherry red spot, infant, progressive familial intrahepatic cholestasis type 1 (pfic1)

## Abstract

Progressive familial intrahepatic cholestasis type 1 (PFIC1) associated with a cherry-red spot, to our knowledge, has never been reported in the literature. We report the case of a 10‑month‑old girl with prolonged cholestasis. A diagnosis of PFIC1 was made by whole‑exome sequencing. Fundus examination showed a cherry-red spot. Our case provides a new insight toward the first case of ocular manifestation of PFIC1. Further studies are required to elucidate FIC1 gene expression in the macula.

## Introduction

Progressive familial intrahepatic cholestasis (PFIC) is an autosomal recessive disorder that causes dysfunction of bile secretion, eventually resulting in end-stage liver disease [1]. Type 1 of PFIC disease is caused by a mutation in the ATP8B1 gene on chromosome 18 that leads to a defect in the familial intrahepatic cholestasis (FIC1) protein which causes non-obstructive cholestasis [2].

A cherry-red spot is a fundoscopy finding visualized with a direct ophthalmoscope. It is a bright red spot at the center of the macula, surrounded and accentuated by a greyish white or yellowish halo. It is a useful sign when clubbed with key clinical features and a good history and often guides the diagnosis of metabolic and neurodegenerative diseases [3].

Patients with PFIC1, often have extrahepatic manifestations during the disease course, such as diarrhea, pneumonia, hearing loss, pancreatic disease, resistance to parathyroid hormone, and growth impairment beyond that attributable to cholestasis [4].

We report the first case of a 10‑month‑old girl presenting with cholestasis found to have PFIC1 associated with a cherry-red spot. A review of the literature is also provided.

## Case presentation

**Figure 1:**
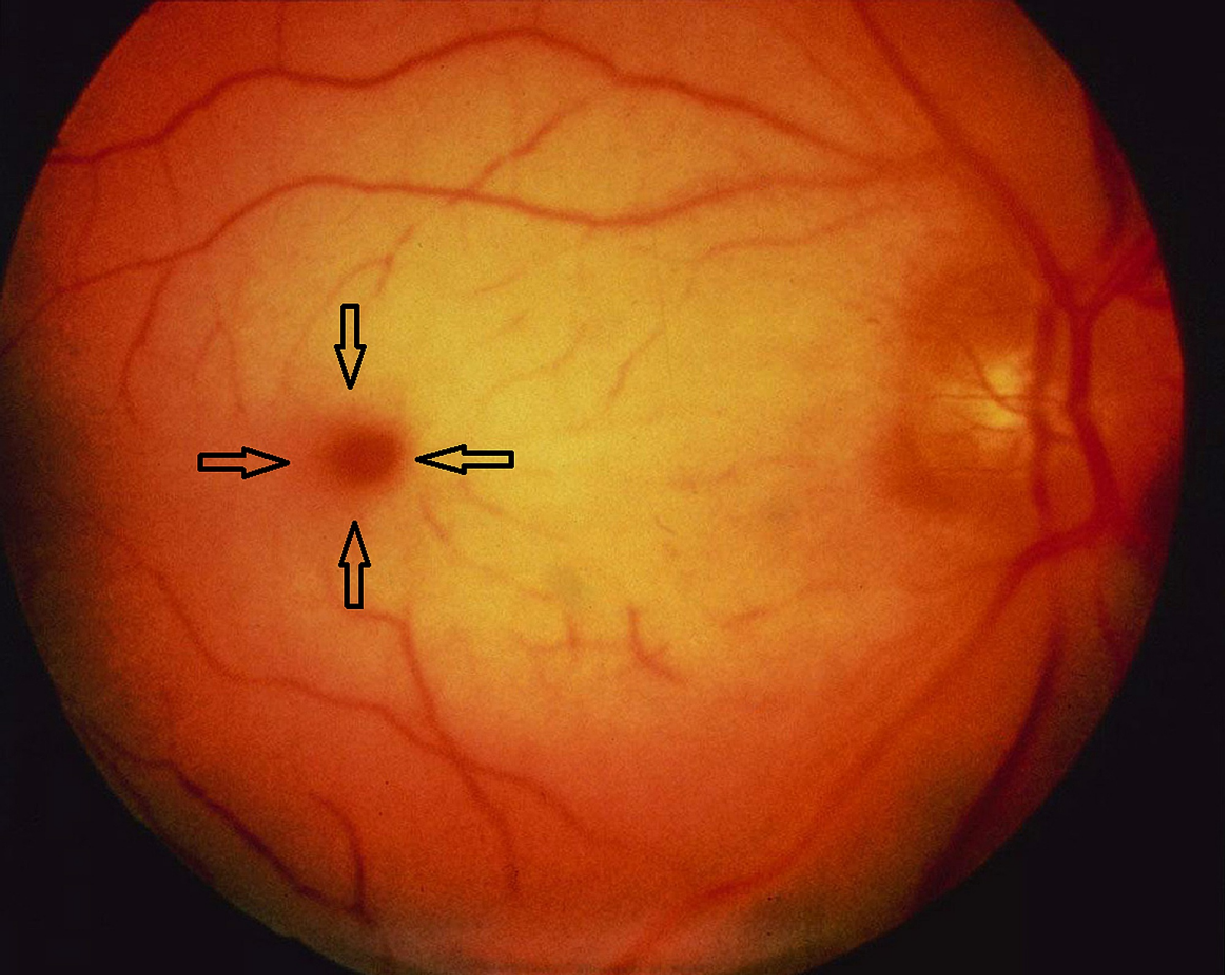
A bright red spot at the center of the macula (arrows), surrounded and accentuated by a greyish white or yellowish halo

A 10-month-old girl was referred to pediatric gastroenterology because of prolonged jaundice for a duration of three months associated with tea-coloured urine and poor weight gain. Also, she had a history of unexplained hypocalcemia and mild developmental delay with a negative history of fever, skin rash, and change in stool color. Past medical history revealed that she was born of a full-term cesarean section delivery and an uncomplicated neonatal course. The parents were first-degree cousins. There was no family history of liver or metabolic diseases. She was not taking medications. Physical examination revealed that her head circumference, weight, and length growth charts were below the third percentile. She looked deeply jaundiced, with dysmorphic features in form of a prominent forehead, deep-seated eyes, small nose, and low-set ears. The abdomen appeared to be soft on examination, along with mild hepatosplenomegaly and ascites. Moreover, an ejection systolic murmur was heard on cardiac examination. The rest of the systemic examination was otherwise unremarkable. Fundus examination showed a reddish area at the center of the macula surrounded by retinal opacification, consistent with cherry-red spots on the maculae of both eyes (Figure 1). Furthermore, the laboratory tests performed on the patient are shown in Table 1. Abdominal ultrasonography reported fullness of the pelvicalyceal system and no evidence of organomegaly or any other abnormalities. Brain MRI showed diffuse abnormal white matter signal intensity.

**Table 1:** Laboratory Results

Test	Result	Normal Range
Platelets	505 x 10^9/L	150 - 450 x 10^9/L
Haemoglobin	11.2 mg/dL	10.6 - 14.5 mg/dL
Total bilirubin	146.2 umol/L	0.8 - 11.7 umol/L
Direct bilirubin	110.90 umol/L	0.8 - 5.2 umol/L
Bile acids	160.4 umol/L	≤ 6.0. umol/L
Alkaline phosphatase	1056 U/L	134 - 518 U/L
Gamma-glutamyl transferase (GGT)	13 IU/L	8 - 127 IU/L
Aspartate transaminase (AST)	71 IU/L	20 - 67 IU/L
Alanine transaminase (ALT)	76 U/L	5 - 33 U/L
Ammonia	65 umol/L	21 - 50 umol/L
Lactate dehydrogenase (LDH)	455 U/L	163 - 452 U/L
Partial thromboplastin time (PTT)	43 sec	26 – 41 sec
International normalized ratio (INR)	1	0.8 - 1.2
Adjusted calcium	1.85 mmol/L	2.25 - 2.75 mmol/L
Parathyroid hormone (PTH)	460.60 pg/mL	6.42 - 88.58 pg/mL
Total 25-hydroxyvitamin D	< 20 nmol/L	50 - 125 nmol/L
Thyroid-stimulating hormone (TSH)	7.47 mIU/L	0.70 - 4.17 mIU/L
Free T4	10.88 pmol/L	11.00 - 20.60 pmol/L

The patient was admitted to the hospital for investigation with a diagnosis of cholestasis. Biliary obstruction, autoimmune and infectious diseases were ruled out. Furthermore, an extensive workup for causes of the cherry-red spot was carried out and the following were excluded: Tay Sach’s disease, Sandhoff disease, gangliosidosis, metachromatic leukodystrophy, Niemann Pick disease, Farber’s disease, Goldberg’s syndrome, Gaucher’s disease, and Hurler’s syndrome. Additionally, a whole-exome sequencing (WES) test was performed, and it identified a homozygous pathogenic variant in the ATP8B1 gene. Considering the ATP8B1 gene mutation and the patient’s phenotype, a diagnosis of PFIC1 was made.

The patient was started on fat-soluble vitamins (A, D, E, K), 1 mL once daily, ursodeoxycholic acid, 15 mg/kg/day, calcium carbonate, 74.6 mg/kg/day, and cholecalciferol, 1000 IU once daily. Because of refractory pruritis, rifampicin, 15 mg/kg/day, and cholestyramine, 240 mg/kg twice daily, were added. At the follow‑up examination when she was 12 months of age, she had gained weight and her cholestasis was stable. Lastly, She was listed for a liver transplant at the age of 24 months.

## Discussion

This case is the first case report of extrahepatic manifestation of PFIC1 associated with cherry-red spots. Pathogenesis of PFIC1 is primarily caused by a defect in the FIC1 protein due to a mutation in the ATP8B1 gene on chromosome 18 [1]. This defect leads to bile acid accumulation in the hepatocytes, rendering it from being secreted to bile canaliculi [5]. Typically, PFIC1 occurs during the first year of life. Patients usually present with jaundice, icterus, severe pruritus, and hepatomegaly, along with several extrahepatic manifestations that are unique to PFIC1 [6]. They include pancreatitis, sensory neural deafness, and short stature [6]. In addition to the common phenotype, high serum bile acids, normal gamma-glutamyl transferase (GGT), and deficiency in fat-soluble vitamins, clinical diagnosis is confirmed by genetic testing. Despite medical treatment, patients eventually develop end-stage liver failure. Thus, a liver transplant is the only definitive treatment [2].

A cherry-red spot occurs when the retinal artery is blocked, causing pallor in the canter of the retina, while the blood supply from the ciliary artery continues to the choroid and results in a bright red discoloration in the macula [7]. This process, if left untreated, will lead to a decrease in visual acuity [7]. On the other hand, it can also arise from the deposition of multiple lipids in the ganglion cell layer leading to the retina becoming opaque except the fovea because it lacks ganglion cells [8]. Therefore, the fovea remains transparent showing the red color of the choroid. Such deposition of lipids occurs in lipid storage disorders, such as Niemann-Pick disease, Farber disease, and metachromatic leukodystrophy.

Cherry red spot has also been reported in quinine, carbon monoxide, methanol, and dapsone toxicity [9]. Our patient did not receive any toxic medications.

This case has the first ocular finding of cherry red spots on both maculae which have not been associated previously with PFIC1. We suggest in our hypothesis that the FIC1 gene is expressed in a variety of tissues, not only the liver, pancreas, and intestine [10]. Thus, this hypothesis needs to be tested in the future. Finally, other systems, such as the eye, should be examined and investigated to rule out extrahepatic manifestations.

## Conclusions

This is the first case report of a cherry-red spot as an ocular manifestation of PFIC1. We suggest considering a detailed eye examination as part of the PFIC evaluation to avoid its consequences. This case provides a new insight for understanding possible FIC1 gene expression in the maculae. Further studies are required to elucidate the pathogenesis of ocular manifestations in PFIC1.
